# Mechanism for the Structural Transformation to the Modulated Superconducting Phase of Compressed Hydrogen Sulfide

**DOI:** 10.1038/s41598-019-41607-1

**Published:** 2019-03-22

**Authors:** Arnab Majumdar, John S. Tse, Yansun Yao

**Affiliations:** Department of Physics and Engineering Physics, University of Saskatchewan, Saskatoon, Saskatchewan S7N 5E2 Canada

## Abstract

A comprehensive description of crystal and electronic structures, structural transformations, and pressure-dependent superconducting temperature (*T*_*c*_) of hydrogen sulfide (H_2_S) compressed from low pressure is presented through the analysis of the results from metadynamics simulations. It is shown that local minimum metastable crystal structures obtained are dependent on the choice of pressure-temperature thermodynamic paths. The origin of the recently proposed ‘high-*T*_*c*_’ superconducting phase with a modulated structure and a diffraction pattern reproducing two independent experiments was the low pressure *Pmc*2_1_ structure. This *Pmc2*_1_ structure is found to transform to a *Pc* structure at 80 K and 80 GPa which becomes metallic and superconductive above 100 GPa. This structure becomes dynamically unstable above 140 GPa beyond which phonon instability sets in at about a quarter in the Γ to Y segment. This explains the transformation to a 1:3 modulation structure at high pressures proposed previously. The pressure trend of the calculated *T*_*c*_ for the *Pc* structure is consistent with the experimentally measured ‘low-*T*_*c*_ phase’. Fermi surface analysis hints that pressurized hydrogen sulfide may be a multi-band superconductor. The theoretical results reproduced many experimental characteristics, suggesting that the dissociation of H_2_S is unrequired to explain the superconductivity of compressed H_2_S at any pressure.

## Introduction

Recent discovery of a very high superconducting temperature *T*_c_ (203 K) in hydrogen sulfide (H_2_S) compressed to 200 GPa by Drozdov *et al*.^1^ stimulated intense investigation. At the moment of this discovery, however, the particular compound responsible for the ‘high *T*_*c*_ phase’ was unknown. Based on the similarity of the measured *T*_*c*_ to an earlier theoretical estimate for H_3_S^2^, Drozdov *et al*. suggested that the phase responsible for the high-*T*_*c*_ superconductivity in compressed hydrogen sulfide may be H_3_S. The H_3_S was proposed as a decomposition product of H_2_S at high pressures, which has a bcc structure (*Im*-3*m*) in the pressure range of interest. Theoretically, the H_3_S is calculated to be the most thermodynamically stable stoichiometry of sulfur hydrides in this pressure range^3–5^. The suggestion is supported by a subsequent experiment^6^ in which both the electrical conductivity and x-ray diffraction (XRD) pattern were measured simultaneously on the compressed H_2_S samples prepared following a *P–T* path similar to that used in the original superconductivity investigation^1^. The observed XRD pattern in the second experiment was interpreted as a mixture of the *Im-3m* structure of H_3_S and β-Po sulfur. In the mixture, the H_3_S is considered the dominant superconducting phase while sulfur is a minor impurity. However, several follow-up experiments to synthesize H_3_S from different thermodynamic compression paths have led to different products which were assigned to various H_x_S species^7,8^. Moreover, Gordon *et al*.^9^ pointed out that the proposed decomposition of H_2_S to H_3_S and S with a 1:2 S to H ratio is inconsistent with the measured XRD pattern of the ‘high-*T*_*c*_ phase’ in which the intensity due to sulfur is much weaker than expected. Recently, Goncharov *et al*.^10^ attempted a direct synthesis of H_3_S by reacting elemental sulfur and hydrogen at high pressures and found that the XRD pattern of the product is similar to that of the compressed H_2_S^6^. It is somewhat surprising that the two weak peaks on either side of the lowest angle Bragg reflection were observed in the XRD pattern at similar *d*-spacing and relative intensities in both experiments^6,10^. Since the synthetic pathways and starting compounds were completely different in the two experiments, the persistent presence of these weak reflections in the XRD patterns are not by coincidence, thus raising a question on the origin. It should be noted that Guigue *et al*.^11^ have also performed a direct synthesis of H_3_S from elemental materials but only obtained an orthorhombic *Cccm* structure up to 160 GPa, rather than the suggested *Im*-3*m* structure. The *Cccm* structure contains H_2_ quasimolecules and it is not metallic, nor superconducting^2^.

The discrepancies in the observed crystal structures obtained from different thermodynamic *P-T* pathways raised the possibility that the superconducting ‘high-*T*_*c*_ phase’ could be from a kinetically controlled metastable product and may not be the predicted H_3_S. To study the kinetic effect, the potential energy landscape of hydrogen sulfide needs to be examined. Metadynamics simulation at finite temperature and pressure is an appropriate technique^12,13^. Previously, we studied an idealized model of H_2_S^14^ based on the non-dissociated zigzag structure proposed by Gordon *et al*.^9^, with the S atoms forming a body-center cubic lattice as much as the experimental XRD pattern reveals. It was found that H_2_S does not decompose into H_3_S and S at the pressure range of interest. Instead, it transforms to a modulated structure with calculated diffraction pattern matching well with that of the observed ‘high-*T*_*c*_ phase’^6^. In the present study, we report results of *ab initio* metadynamics calculations performed at a broad *P–T* regime to trace the transformation pathway from the low-pressure H_2_S precursor to the high pressure modulated structure. In particular, we identified a stoichiometric *Pc* structure with estimated *T*_*c*_ following the observed trend of the long-sought ‘low-*T*_*c*_ phase’. Theoretical calculations show that the transverse acoustic phonon modes in the *Pc* structure start to soften near 140 GPa which gradually develop to an imaginary branch near the boundary of the Brillouin zone (BZ) at higher pressures. The phonon softening appears in a particular fashion which is consistent to a reconstruction of the supercell along the lattice vector direction leading to a 1:3 modulation of the unit cell. The *T*_*c*_ of the modulated structure is estimated to be within 110–220 K at 200 GPa, which compares well to the measured *T*_*c*_ for the ‘high-*T*_*c*_ phase’. Therefore, the *Pc* structure is likely to be the underlying structure along the experimental compression path.

## Results

**Figure 1:**
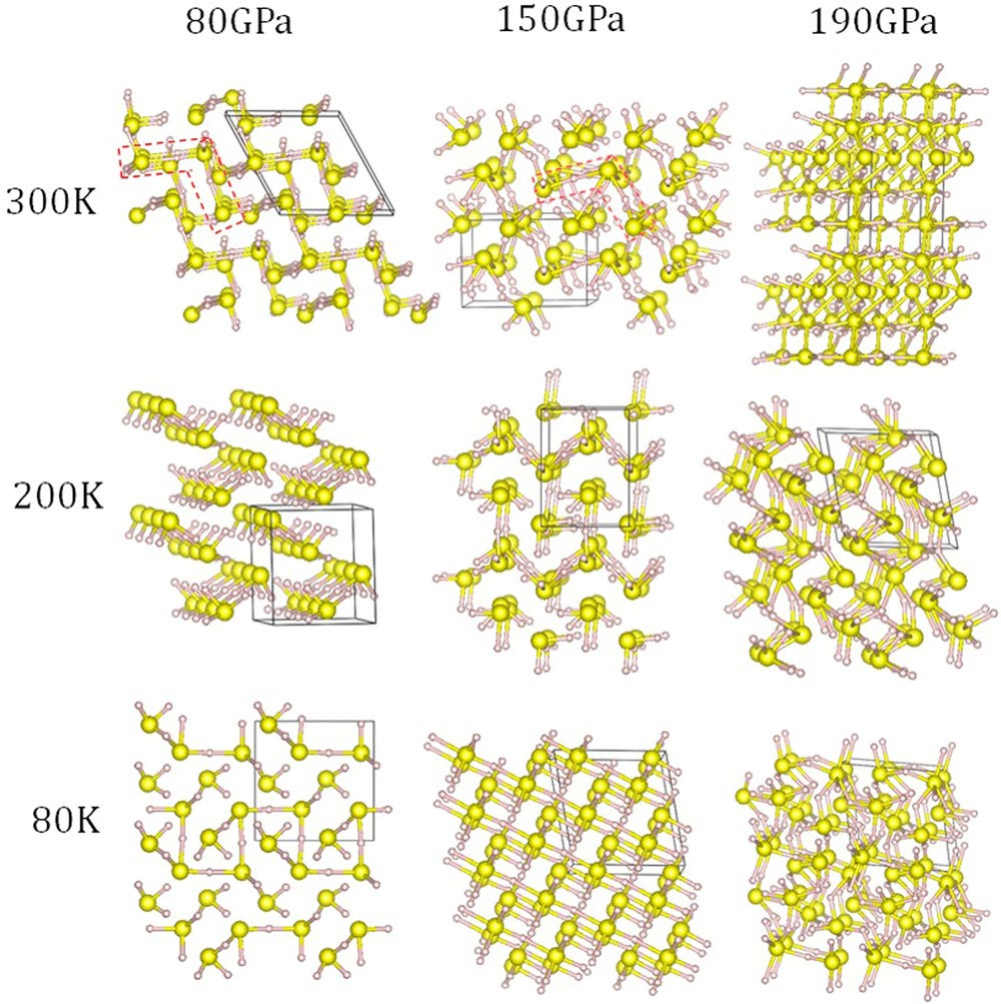
Crystal structures of H_2_S obtained from metadynamics simulations carried out at different pressures and temperatures, starting from the *Pmc*2_1_ structure. Big and small spheres represent S and H atoms, respectively. Unit cells are marked with black boxes.

In an earlier theoretical prediction on high-pressure H_2_S structures, a polymeric *Pmc*2_1_ structure was found to be the most thermodynamically stable between 65 and 80 GPa^15^. The simulated XRD pattern of the *Pmc*2_1_ structure also matches well with the measured phase (phase V) in this pressure range^16^. We therefore chose this structure as the precursor for metadynamics simulation and performed the simulation in a broad *P–T* regime to explore the structural phase transition sequence and mechanisms. In these simulations, reconstructive phase transitions are identified by consecutive drops in the average enthalpy of the supercell which evolves with time. From the simulation results, the first thing one notices is that the structural morphologies of the metastable H_2_S phases identified along the 80 K, 200 K and 300 K isotherms at 80 GPa, 150 GPa and 190 GPa starting from the same precursor are very different (Fig. 1). This reveals the changes in the potential energy surfaces of compressed H_2_S at different *P–T* points.

At 80 K and 80 GPa, the *Pmc*2_1_ structure transforms to a structure composed of zigzag SH chains and H_3_S quasimolecules. In this structure, valence electrons are partially transferred from the H_3_S to the SH forming nominally SH^−^ and H_3_S^+^. The electrostatic attractions between the two species provide stability to the structure. In contrast, on increasing the temperature to 200 K, the extended chain structure does not form. The H_2_S is then a ‘molecular crystal’ consisting of H_2_S-HSH quasimolecules. The H_2_S-HSH has electrostatic polarity in which one H_2_S is cationic and the other is anionic. The intermolecular interactions in the crystal are primarily hydrogen bonds (H…S−H) and secondary dihydrogen bonds (S−H^δ−^… H^δ+^−S)^17^. At 300 K, longer, zigzag quasimolecules are developed in the solid (outlined by the red dash box). Clearly, reducing the temperature enhances the intermolecular interactions and drives the crystal structure to a higher dimensionality, from 0D molecule to 1D chain. One expects similar change of dimensionality when the pressure is increased. At 150 GPa and 80 K, no molecular species can be retained in the structure and only linear S-H chains are observed. At 200 K, the H_2_S-HSH quasimolecules formed at the same temperature but low pressure (80 GPa) are now linked to a 3D extended framework. At 300 K, reminiscent of the dimerized H_2_S-HSH units is still evident but the molecular units are more densely packed. At 190 GPa and 80 K, a 3D framework with S-H-S linkages is formed. The structure is stabilized by electron-deficient multicenter S-H-S interactions when neighboring sulfur atoms are linked together through a common hydrogen atom. Within the linkage, electrons are delocalized and this leads to a metallic state, possibly also a superconducting state, for H_2_S (see later). A very similar morphology is observed at 200 K. However, at 300 K, a 3D framework built solely of S atoms is formed in which the S atoms are directly bonded to each other rather than bypassing a hydrogen atom. The dissociation of the S-H-S bonds suggests the onset of the phase segregation near the dissociation point.

The results obtained from metadynamics simulations show that the structures of H_2_S are sensitively dependent on the *P-T* condition of compression. High temperature is required to overcome the activation barrier in breaking the S-H bonds and to dissociate molecular H_2_S into segregation of elemental S and H_x_S species. It is therefore possible that the compound responsible for the experimental ‘high-*T*_*c*_ phase’^1^ was determined by the choice of the low-temperature compression path in which the theoretical thermodynamic stable phase (*i.e*., *Im*-3*m* phase of H_3_S)^2^ may not form. This is a reasonable scenario to explain the very different diffraction patterns observed for the samples compressed along different *P-T* paths^10,11^. Of all the structures obtained, the zigzag chain structure at 80 GPa and 80 K (Fig. 1) is the most interesting one since it resembles the modulated structure reported earlier^14^. Once fully optimized, this structure has a monoclinic unit cell in the *Pc* space group. In this structure the S atoms form a distorted bcc lattice with three H atoms bonded to each body-centered S and forming an H_3_S cation. The remaining H atoms are located between the corner S atoms, forming extended (SH)_∞_ chains. This structure is obviously the precursor of the high pressure modulated (SH)^−^ (H_3_S)^+^ structure. On the other hand, the structures obtained at 150 and 190 GPa are either unstable or energetically unfavorable since the starting structure (*Pmc*2_1_) already becomes unstable at these pressures with soft phonon modes occurring in some regions of the BZ. The details of these structures will not be analyzed further.

**Figure 2:**
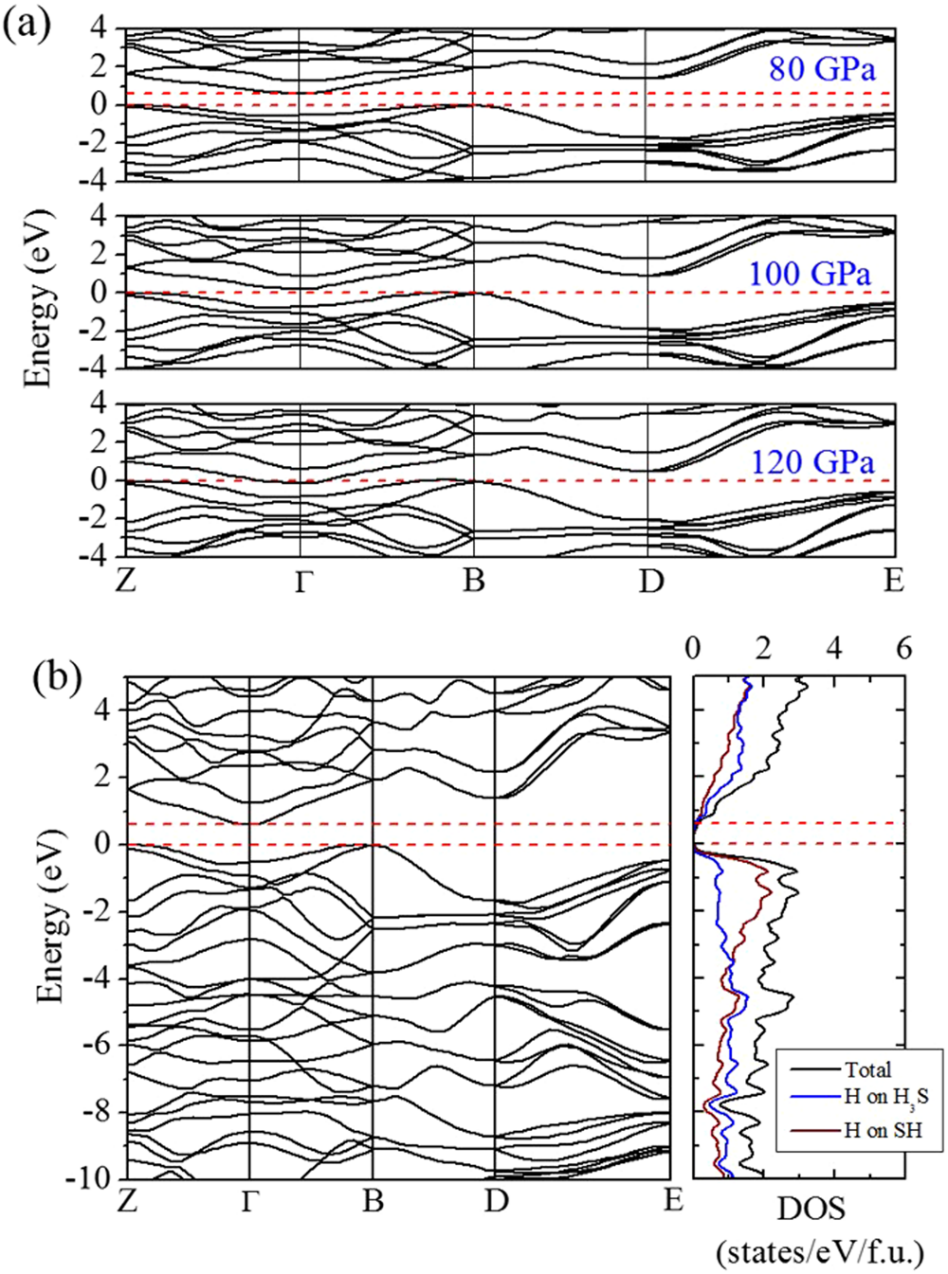
(**a**) Evolution of the electronic band structure of the *Pc* structure near the Fermi level at 80, 100 and 120 GPa. (**b**) Electronic band structure and projected DOS of the *Pc* structure at 80 GPa. The Fermi level and band gap are indicated by dash lines.

**Figure 3:**
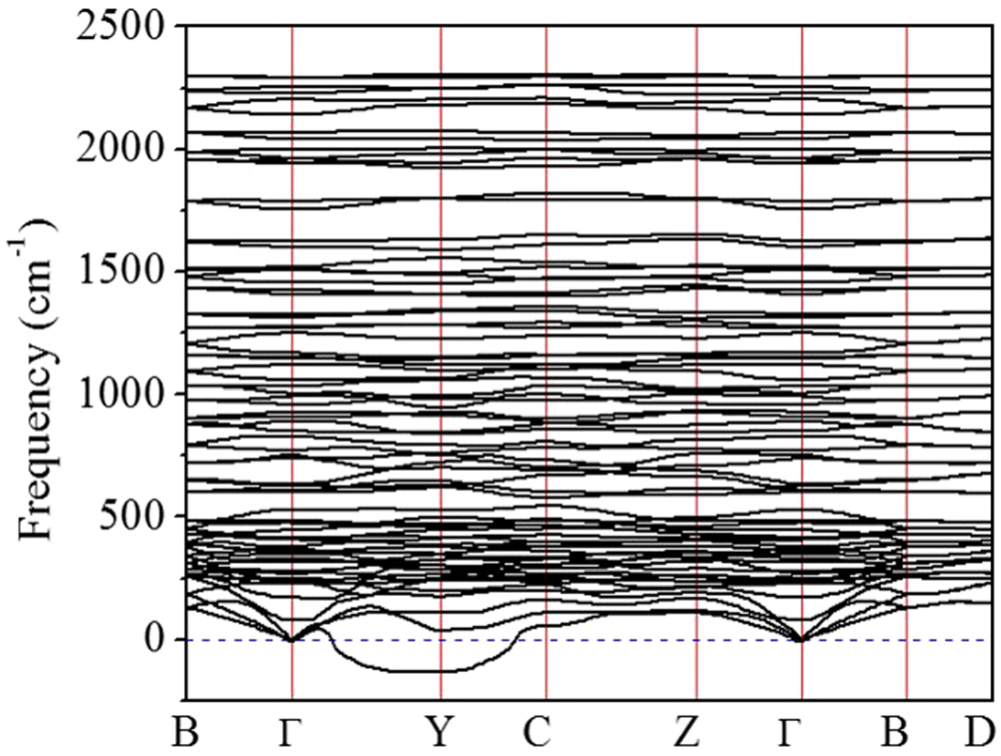
Phonon dispersion curves of the *Pc* structure curve at 180 GPa.

To understand how the *Pc* structure evolves to the modulated (SH)^−^ (H_3_S)^+^ structure at high pressures, we investigate the mechanical and electronic properties of this structure at selected pressures. At 80 GPa, the *Pc* structure has a semiconducting ground state with a band gap of about 0.6 eV (Fig. 2a). The calculated electronic density of states (DOS) shows that the S-H chains contribute more than the H_3_S cations to the states near the band gap (Fig. 2b). The small band gap is partially due to the unsymmetrical S-H-S bonds along the chain (H is slightly off the center). In the conduction band (consisting of S-H antibonding states), contributions from both the chain and the cation S atoms are almost equal. At 100 GPa, the band gap is closed due to the indirect overlap of the valence and conduction bands. At 120 GPa, the lowest-energy conduction band crosses the Fermi level and develops an electron-pocket near Γ. Therefore, the *Pc* structure becomes metallic at pressures higher than 100 GPa. The delocalization of the electrons in the *Pc* structure upon increasing pressure induces softening in the transverse acoustic phonon branches starting at 140 GPa, gradually developing into imaginary frequencies at higher pressures. At 180 GPa, the acoustic vibrational bands with imaginary frequencies are located about three quarters of the phonon vectors along the Γ → Y and C → Y symmetry directions (Fig. 3). The appearance of the imaginary modes is consistent with the reconstruction of the supercell into 1:3 ratio modulation along the lattice vector direction similar to the structure found in the molecular dynamics simulations^14^. Thus, the formation of modulated structures at high pressures is driven by the intrinsic instability of crystalline hydrogen sulfide. The inclusion of aharmonicity corrections is likely to modify the predicted phase transition pressures as shown, for example in H_3_S^2,5^, but should not alter the sequence of the transitions.

**Figure 4:**
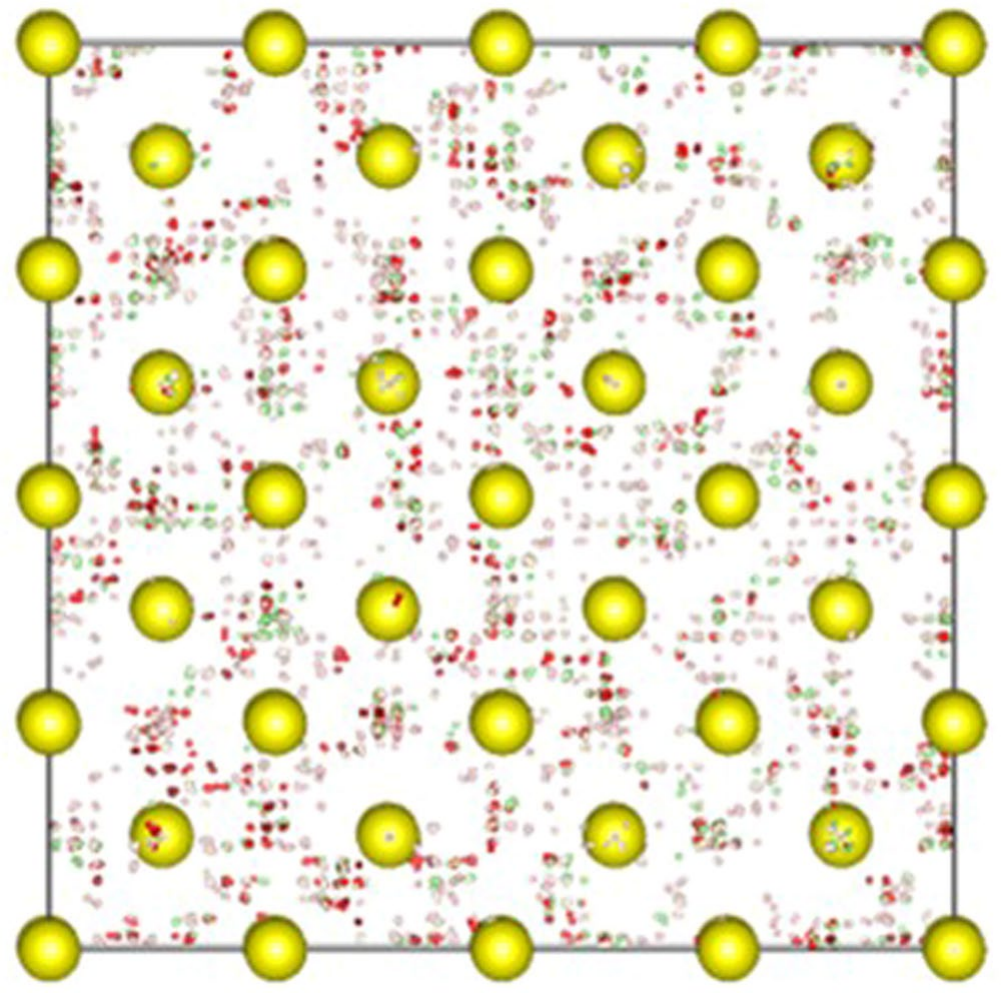
Temporal positions of the S and H atoms indicated by different colors at different slices from molecular dynamics simulation (see text) showing the rapid hopping and diffusing motion of the hydrogen atoms at 160 GPa.

The instability of H_2_S at high pressure is revealed in first principles molecular dynamics (FPMD) simulations performed in a canonical (*NVT*) ensemble on a model constructed of a bcc lattice with the internal atomic positions mapped from the *Pc* structure. At 120 GPa, all of the H atoms in the structure vibrate about the respective atomic sites and maintain the basic zigzag chain and H_3_S moieties. When the pressure is increased to 160 GPa, however, the motions of the H atoms become highly mobile which smear out the identity of the chain and molecules. Once the temporal positions of the H atoms are color-coded according to different time segments of the FPMD trajectory (Fig. 4), it is quite obvious that there are collaborative hopping and diffusing motions between different H sites. In this situation, the hydrogen atoms do not have fixed atomic positions; the structure may be described by a bcc lattice of sulfur filled by fluxional hydrogen atoms. The high-mobility H motions substantially softened the vibrational modes and altered the long-range order in the structure. It is should be noted that in the *NVT* ensemble, the shape and size of simulation cell is fixed so the sulfur atoms are more or less kept at the lattice sites by this constraint. If the unit cell vectors are allowed to evolve under external stress, a modulation of the supercell will occur following the soft phonon modes. In the previous isothermal–isobaric ensemble (*NPT*) FPMD simulation^14^, a 1:3 body centered tetragonal modulated structure was developed at 200 GPa and 200 K for which the modulation vector agrees well with the eigenvectors of the soft phonon modes (Fig. 3).

Superconductivity was observed in compressed H_2_S at pressures as low as 110 GPa. Referred to as the ‘low-*T*_*c*_ phase’, this superconducting phase is achieved by cold compressing (at around 100 K) the H_2_S sample to high pressures^1,6^. The *T*_*c*_ of the ‘low-*T*_*c*_ phase’ spans between 33 and 150 K when the pressure is increased from 110 to 200 GPa. The other superconducting phase, ‘high-*T*_*c*_ phase’, is obtained by annealing the H_2_S sample compressed at 200 K. The ‘high-*T*_*c*_ phase’ has notably higher *T*_*c*_, which reaches the maximum of 203 K at 155 GPa and then gradually decrease with pressure^1,6^. Experimentally, little is known about the chemical composition and structure of the ‘low-*T*_*c*_ phase’. Theoretically, many structure models based on thermodynamic stability consideration and estimation of *T*_*c*_ have been proposed^7,15,18–21^. In particular, a modulated structure model with long periods of alternating slab-like H_2_S and H_3_S regions forming a sequence of ‘Magnéli phase’ with variable stoichiometries H_*x*_S_1−*x*_ (2/3 < *x* < 3/4)^18^ seems to be able to produce the trend of change in *T*_*c*_ for the ‘low-*T*_*c*_ phase’. In principle, the *T*_*c*_ of the ‘Magnéli phase’ may be tuned, if one can adjust the H_2_S:H_3_S ratio in the structure, to range from the lowest value with pure H_2_S to the highest value with pure H_3_S. Even though the pressure trend of a comparatively low *T*_*c*_ may be reproduced, the calculated XRD patterns of these ‘Magnéli phases’ do not match to the experimental data^22^.

The theoretical results presented above show unambiguously that there is a direct connection between the low-pressure phase V (*Pmc*2_1_) and high-pressure modulated structure *via* the intermediate *Pc* structure. The question is whether the *Pc* structure in this pressure region is superconductive and, if so, would the *T*_*c*_ also agree with the experimental observations for the ‘low-*T*_*c*_ phase’? For this purpose, we need to investigate the superconducting properties of the *Pc* structure in stable pressure range. However, quantitative prediction of the *T*_*c*_ from first principles for a modulated and hydrogen disordered system is difficult. The *T*_*c*_ values in hydrogen sulfide phases have been computed by different groups using essentially the same methodology of strong coupling Migdal-Eliashberg formulism^23,24^ based on the Bardeen-Cooper-Schrieffer (BCS) theory^25^. Although the electron-phonon coupling (EPC) strength *λ* may be calculated at a deeper level of theory, *T*_*c*_’s are often estimated from approximations, such as the assumption of single effective band, constant DOS, and isotropic pairing, as well as the lack of first principles treatment of Coulombic pseudopotential (μ*)^2,5,19,26,27^. Therefore, the theoretical *T*_*c*_’s obtained by these methods only provide a consistency check, rather than the unequivocal confirmation of the experimental results. Furthermore, the *T*_*c*_ for the ‘high-*T*_*c*_ phase’ has not been established firmly from experiment. The values obtained from resistivity measurement^1^ and those from the measurement of Meissner effects^28^ can differ by several tens of Kelvin. Under this circumstances, the uncertainties in the *T*_*c*_ in both experiment and theory should be kept in mind.

Central to the Migdal-Eliashberg theory is EPC parameter *λ* obtained by integrating the electron-phonon spectral function *α*^2^*F*(*ω*),

1 $$\lambda =2{\int }_{0}^{\infty }\frac{{\alpha }^{2}F(\omega )}{\omega }d\omega .$$

In strong coupling region, the *T*_*c*_ can be estimated using the Allen-Dynes modification of the McMillan equation^29^,

2 $${T}_{c}=\frac{{\omega }_{\mathrm{log}}}{1.2}\exp [-\frac{1.04(1+\lambda )}{\lambda -{\mu }^{\ast }(1+0.62\lambda )}],$$

where *ω*_log_ is the logarithmic average of phonon frequencies and μ* is the Coulomb pseudopotential representing the screened Coulombic repulsion.

**Figure 5:**
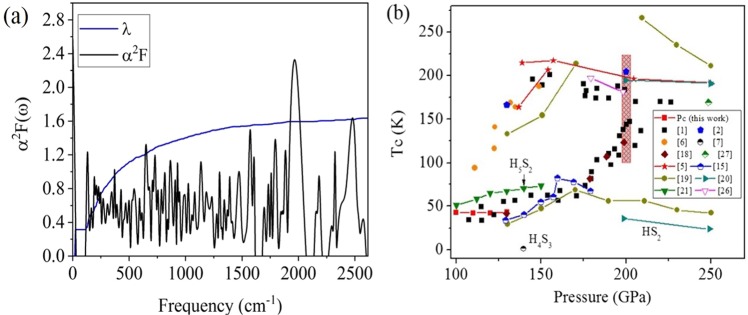
(**a**) The *α*^2^*F*(*ω*) and *λ*(*ω*) of the *Pc* structure calculated at 120 GPa. (**b**) Comparison between the calculated *T*_*c*_ for various H_x_S species (all but refs^1,6^) and the measured *T*_*c*_ in compressed H_2_S^1,6^. The shaded red region indicates the range of estimate for the *T*_*c*_ at 200 GPa for the modulated 1:3 structure.

The electronic band structures of the *Pc* structure (Fig. 2a) reveals simultaneous occurrence of curve (dispersive) and flat (diffusive) bands near the B symmetry point and close to the Fermi level, which is considered a favorable condition for strong electron-phonon coupling^30,31^. The *α*^2^*F*(*ω*) and integrated *λ* for the *Pc* structure calculated at 120 GPa are shown in Fig. 5a, which shows that the EPC is weighted more heavily in the low-frequency region primarily by the S-H-S vibrations in the chains. The obtained *λ* has more than 80% contribution from vibrations below 1000 cm^−1^. The high-frequency S-H vibrons of the H_3_S quasimolecules (above 2000 cm^−1^) contribute very little to the EPC. Up to 1500 cm^−1^ there is strong coupling of H and S vibrational modes. The bands from approximately 500 to 1500 cm^−1^ can be attributed to S-H-S bending modes. This is contrast to what has been found in phosphine (PH_3_) which is an analogue of H_2_S. In an earlier experiment by Drozdov *et al*.^32^, PH_3_ was found to have *T*_*c*_ > 100 K pressures above 200 GPa. Theoretical studies on PH_3_ confirmed this observation^33,34^. Phosphorus hydrides are calculated to be unstable against elemental decomposition up to pressures of 400 GPa. Therefore, it was suggested that the high *T*_*c*_ superconductivity observed in phosphine were purportedly metastable phases^33^. Similar results on the decomposition of phosphine and superconductivity in metastable phases were calculated and presented by Flores-Livas *et al*.^35^ and Shamp *et al*.^36^ But unlike P hydrides, from our calculated structures, H_2_S does not contain any S-S bond. The absence of the S-S vibrations (unlike P-P vibrations in P hydrides) indicates that the electron−phonon interactions leading to a relatively high *T*_*c*_ in each case differ, albeit more effective in H_2_S. The EPC distribution suggests that the superconductivity of the *Pc* structure mainly arises in the S-H-S chains, rather than in the H_3_S molecules. This is corroborated by the band structure which has the states near the Fermi level primarily occupied by the S-H-S chains (Fig. 2b). Using a nominal value of 0.1 for the μ* ^37^, the estimated *T*_*c*_, *ω*_*log*_ and *λ* for the *Pc* structure are shown in Table 1. Remarkably, the *T*_*c*_ is in good agreement with the experimental data for the ‘low-*T*_*c*_ phase’ (Fig. 5b). The *T*_*c*_ remains almost constant from 100 to 130 GPa as the *λ* increases but the *ω*_log_ decreases, which can be attributed to the gradual softening of the S-H-S stretch vibrations when the structure is compressed toward the unstable region. At higher pressures, the S-H stretch and H-S-H bent vibrations are more efficiently mixed in the mid-frequency region which enhances the EPC. Moreover, the DOS at the Fermi level also increases slightly with the pressure as the metallicity of the structure increases. However, overall, the *T*_*c*_ remains almost constant at 42 K since the decreasing *ω*_*log*_ weighs down all other contributions. The estimated *T*_*c*_ of the *Pc* structure are comparable to the reported theoretical values for H_x_S species like H_5_S_2_ and Magnéli phases^18,21^.

**Table 1:** The *λ*, *ω*_log_ and *T*_*c*_ for the *Pc* structure.

P (GPa)	*λ*	*ω*_*log*_ (K)	*T*_*c*_ (K)
100	1.49	380	42
110	1.56	356	42
120	1.64	344	42
130	1.75	332	43

We now shift the discussion to the 1:3 modulated structure obtained previously from the *NPT* FPMD simulation^14^. In this structure, body-centered tetragonal and cubic unit cells are aligned alternatively in a 1:3 ratio, forming a modulation with quadrupled period. This structure is conceptually similar to the ‘Magnéli phase’^18^, but it still maintains a H_2_S stoichiometry. In the 1:3 modulated structure, the H atoms undergo rapid diffusions, and are more mobile in the tetragonal region. The high mobility of the fluxional motion engage the phonons to pair with electrons as per the BCS theory^30^, whereas the large amplitude of the motions leads to the band crossing at the Fermi level. However, without fixed atomic positions numerical estimation of the *T*_*c*_ is very difficult. In what follows we make an order-of-magnitude estimation of the *T*_*c*_ for the modulated structure. At first an *NPT* FPMD simulation is carried out on the modulated structure at 200 GPa and 200 K for 20 ps. The vibrational DOS (*v*DOS) is then obtained from the FPMD trajectory^22^, which captures both harmonic and anharmonic vibrations^38^. Using harmonic approximation^39^, a Debye temperature Θ of 1853 K is estimated from the *v*DOS. The *T*_*c*_ is then estimated using the original McMillan equation^40^,

3 $${T}_{c}=\frac{{\rm{\Theta }}}{1.45}\exp [-\frac{1.04(1+\lambda )}{\lambda -{\mu }^{\ast }(1+0.62\lambda )}].$$

**Figure 6:**
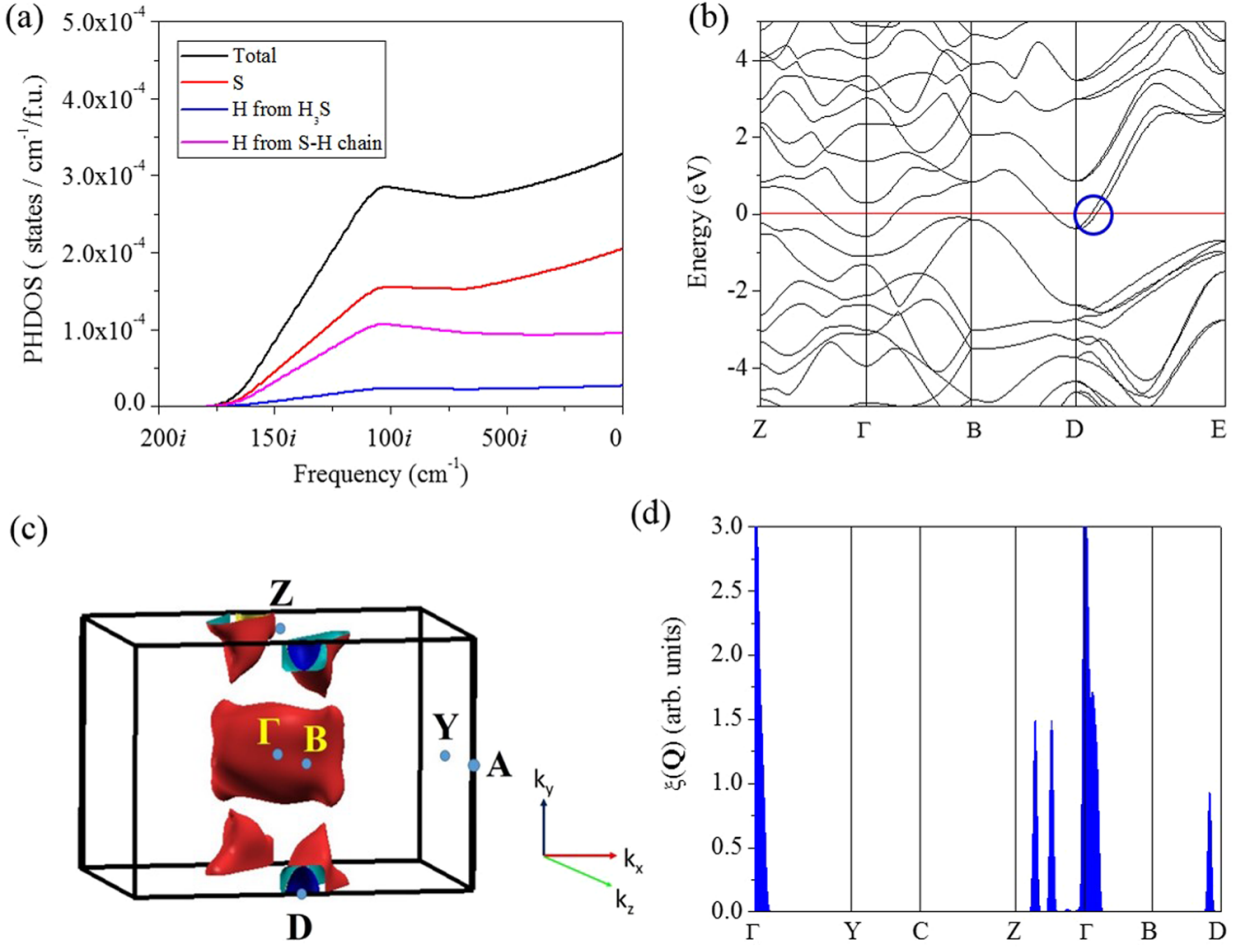
(**a**) Total and atom projected phonon density of states, (**b**) electronic band structure, (**c**) Fermi surfaces, and (**d**) nesting function ***ξ*** (**Q**) for the *Pc* structure at 160 GPa.

Due to the structural modulation, large unit cell, and especially the fluxional motion of the H atoms, it is not feasible to compute the *λ* explicitly. Therefore, we selected several values of *λ* between 1.0 and 2.0, which are within the reasonable range for the *λ* predicted for H_2_S and H_3_S. The estimated values of *T*_*c*_ at 200 GPa are given in Table 2. The estimated *T*_*c*_ for *λ* ≥ 1.5 from 180–220 K are obviously comparable with the experiment for the ‘high-*T*_*c*_ phase’, however, they should be only taken as upper bound estimates since the *λ* has not been obtained quantitatively. Also, the McMillian equation is known to be accurate for all superconductors with λ ≤ 1.5 but in error for large values of λ^29^. Figure 5b represents the phase diagram of the *T*_*c*_ calculated for various H_x_S species and the comparison to the experimental data, from which one may envision that the *Pc* and modulated structures are responsible for the ‘low-*T*_*c*_ phase’ and ‘high-*T*_*c*_ phase’, respectively. The *T*_*c*_ in the ‘low-*T*_*c*_ phase’ has seen a quick rise between 175 and 200 GPa which is likely due to the enhanced electron-phonon coupling near the point of phonon instability^41^. The suggested ‘Magnéli phase’ with continually changing stoichiometry is likely hindered thermodynamically. Above 140 GPa, imaginary phonon branches start to appear in the *Pc* structure. From the projected vibrational DOS (Fig. 6a), these modes are mainly due to the large amplitude motions of the H and S atoms in the S-H-S chains. However, phonon instabilities in harmonic approximation may not indicate the structure immediately at physical instability but it should be close to that. As the acoustic modes soften, atomic displacements are enhanced and the lattice can be stabilized to even stronger coupling by anharmonic interactions. This is exactly what is shown for the *Im*−3*m* structure of H_3_S^5^, and it is entirely feasible for H_2_S as well. Thus, although the phonon modes show instability above 140 GPa, it is possible the *Pc* structure is anharmonic stabilized in a range beyond this pressure and sees a quick rise in *T*_*c*_. Precedent examples of such anharmonic stabilized superconductor include MgB_2_^42^ and compressed Li^43,44^.

**Table 2:** Estimated *T*_*c*_ of the modulated 1:3 structure corresponding to different EPC parameter λ.

λ	1.0	1.2	1.4	1.6	1.8	2.0
*T*_*c*_ (K)	107	137	163	185	204	221

Recently, it has been suggested that the pressurized hydrogen sulfide is a multi-gap material in which the inter-band coupling plays a key role for the emergence of high *T*_*c*_^45,46^. The observed changes in the isotope coefficient under pressure deviate significantly from the prediction of the single-gap Eliashberg theory^47^. It is understood that the phonons only strongly interact with electrons in very restricted regions of momentum space defined by the topology of the Fermi surface. For example, the electronic band structure of the *Im*-3*m* structure of H_3_S undergoes several Lifshitz transitions with new hot spots appearing in the Fermi surface at the pressure coinciding with the onset of the ‘high-*T*_*c*_ phase’^48^. For the *Pc* structure, the band gap closes gradually with increasing pressure (Fig. 2a) and eventually leads to an electronic instability. At 160 GPa, two considerably dispersive parallel bands cross the Fermi level (Fig. 6b) at the Γ and D symmetry points. The band crossings give rise to new Fermi surfaces with small electron pockets around these two symmetry points (Fig. 6c). This change in the Fermi surface is in fact very similar to the reported Lifshitz transition of type one in the *Im*-3*m* structure^48^. The two bands that cross the Fermi level, while in most momentum regions being degenerate, are parallel along the D → E direction (blue circle). The occurrence of parallel bands in the band structure is a sign of possible nesting of the two Fermi surfaces, which can be characterized from the calculation of the nesting function,

4 $$\xi (Q)=\frac{1}{N}\sum _{{\bf{k}}}\delta ({\varepsilon }_{{\bf{k}}})\delta ({\varepsilon }_{{\bf{k}}+{\bf{Q}}})\propto \oint \frac{d{\ell }_{{\bf{k}}}}{|{{\bf{v}}}_{{\bf{k}}}\times {\bf{v}}{}_{{\bf{k}}+{\bf{Q}}}|}.$$

Here the integral follows the intersection of Fermi surface (*E*_*F*_ = 0) and its image displaced by vector **Q**. The *ε*_**k**_ and **v**_**k**_ are the Kohn-Sham eigenvalue and velocity, respectively, and *N* is the number of *k* points. The ***ξ*** (**Q**) determines the possibility of two electrons presenting at the Fermi surface with small and/or collinear velocities (**v**_**k**_ and **v**_**k+Q**_), a necessary condition for coupling two electrons as per the BCS theory. Kohn anomaly is a special case of nesting with a nesting vector **Q** of 2**k**_*F*_.

The ***ξ*** (**Q**) calculated for the *Pc* structure at 160 GPa is shown in Fig. 6d. Interestingly, it does show localized peak-like distribution in the BZ. The large peak around the Γ point (**Q** = 0) is manifestation of the nesting between two degenerated bands and that between the Fermi surface and itself (which obviously has no physical significance). The calculated peaks observed along the Γ-Z and B-D directions indicate that considerable portions of the Fermi surface are nested by two **Q** vectors, (0, *y*, 0) and (0, *y*, 1/2), where 0 < *y* < 1/2. Incidentally, the direction of one of the **Q** vectors coincides with the phonon softening mode along the Γ-Y direction (Fig. 3). An important message obtained from the ***ξ*** (**Q**) is that there are strong and localized nestings in H_2_S primarily due to interband interactions. This observation indicates that hydrogen sulfide may indeed be a multiband superconductor where the interband coupling contribute substantially to the *T*_*c*_^49^. In general, the occurrence of a Fermi surface nesting indicates that the structure is at the proximity of electronic instability. Simply, the linewidth of phonon mode (**q** *j*) arising from electron-phonon coupling is^50^,

5 $${\gamma }_{{\bf{q}}j}=2\pi {\omega }_{{\bf{q}}j}\sum _{nm}\int \frac{{d}^{3}k}{{{\boldsymbol{\Omega }}}_{BZ}}|{g}_{{\bf{k}}n,{\bf{k}}+{\bf{Q}}m}^{j}{|}^{2}\delta ({\varepsilon }_{{\bf{k}}n})\delta ({\varepsilon }_{{\bf{k}}+{\bf{Q}}m}),$$

by virtue of averaging nesting functions weighted by electron-phonon matrix element ${g}_{{\bf{k}}n,{\bf{k}}+{\bf{Q}}m}^{j}{\rm{.}}$ Obviously, the linewidth can be decomposed into two parts, *i.e*., the probability of two electrons coupled through a particular phonon mode, and secondly the strength of this coupling.

It is noteworthy that the calculated *v*DOS of the modulated 1:3 structure^14^ shows a key feature common to solid hydrogen and metallic hydrides with high *T*_*c*_ (*e.g*. YH_6_)^51^ in which there are no longer distinctive separations between the lattice, H-bent and H-stretch vibrational bands. Once the H-bent and H-stretch modes are mixed, all vibrational modes are expected to contribute to electron-phonon coupling and a high *T*_*c*_ may be achieved.

## Discussion

In summary, the electronic structure, lattice dynamics, and electron-phonon coupling of a new *Pc* structure of compressed hydrogen sulfide revealed from metadynamics calculations were presented and discussed in detail. We show that the *Pc* phase, which evolves from the low-pressure polymeric precursor, follows a low temperature compression path starting at 80 GPa eventually to a modulated structure at 200 GPa. The latter transformation is due to the phonon instability near ¼ along the Γ→ Y and C → Y symmetry directions at pressures above 140 GPa which leads to a commensurate 1:3 modulation. The *Pc* structure is calculated to be superconducting between 100–130 GPa. The estimated superconducting transition temperature *T*_*c*_ ~ 42 K is not sensitive to the pressure in this pressure range. An order of magnitude estimate of the *T*_*c*_ for the 1:3 modulated structure from the calculated Debye temperature at 200 GPa is within 110–220 K, which is in reasonable agreement with the experimental observation. The finding of a direct structural connection between the low-pressure polymeric structure to the ‘high-*T*_*c*_’ superconducting phase as well as reasonable superconductivity estimated for the ‘low-*T*_*c*_’ region provide strong evidence to support the alternative explanation that sulfur hydrides with different stoichiometry other than H_2_S are not needed to rationalize the structure and superconductivity of highly compressed H_2_S in the entire pressure range. Fermi surface topology analysis provides evidence that pressurized hydrogen sulfide may be a multi-band superconductor, which welcomes new experimental and theoretical investigations in this direction.

## Methods

First principles enthalpy and electronic structure calculations were performed using the Vienna *ab initio* Simulation Package (VASP) program^52^ version 5.3.3 with projector augmented wave (PAW) potentials^53^. The Generalized Gradient Approximation (GGA) for the exchange-correlation functional parameterized by Perdew–Burke–Ernzerhof (PBE)^54^ was used. A (16 × 16 × 16) Monkhorst Pack (MP) grid^55^ was used for the sampling and integration of the Brillouin zone (BZ). The kinetic energy cut-off of the plane waves is 280 eV. Metadynamics simulations were performed in the pressure range 80–200 GPa, at selected temperatures between 80–300 K. The scaled components of the edge vectors of the simulation supercells were used as collective variables^12,13^. The VASP code was used for first principles molecular dynamics (FPMD) in the isothermal-isobaric (*NPT*) ensemble with Langevin dynamics and in the isothermal-isochoric (*NVT)* ensemble. Phonon calculations were performed using the Quantum ESPRESSO package^56^ version 5.4.0 with norm-conserving pseudopotentials and an energy cut-off of 80 Ry. Dynamical matrices were calculated on a 4 × 2 × 2 *q*-point mesh with an 8 × 8 × 8 *k*-point mesh for the BZ sampling. The electron-phonon coupling (EPC) parameter and logarithmic average of the phonon frequencies obtained between 100 to 130 GPa were calculated within the framework of the BCS theory^25^. To evaluate the linewidth (double delta equation), the Methfessel-Paxton smearing was employed.

## Data Availability

Data available on request from the authors.
